# Cu-Based Multicomponent Metallic Compound Materials as Electrocatalyst for Water Splitting

**DOI:** 10.3389/fchem.2022.913874

**Published:** 2022-06-13

**Authors:** Peijia Wang, Jingjing An, Zhenyu Ye, Wei Cai, Xiaohang Zheng

**Affiliations:** School of Materials Science and Engineering, Harbin Institute of Technology, Harbin, China

**Keywords:** Cu-based materials, electronic structure, water splitting, valence states, multicomponent

## Abstract

In this study, Cu-based multicomponent metallic compound materials M-Cu (M = Mn, Fe, Co, Ni, and Pt) were studied as electrocatalytic materials for water splitting. Different metal materials attached to the copper foam substrate can change the valence states of copper and oxygen, resulting in the change of electronic structure of the materials, thus changing its catalytic activity.

## Highlights

• The M-Cu (M = Mn, Fe, Co, Ni, and Pt) samples were obtained by a facile method on the copper foam substrate.
• The good HER and OER performances come from the introduction of the second ions, which change the valence states of copper and oxygen elements, resulting in the change of electronic structure of the materials.

## Introduction

Electrochemical processes such as water splitting are a promising method to alleviate energy and environmental problems (Zhang et al., 2017a; Anandhababu et al., 2018; Zhang et al., 2019a). However, the efficiency of anodic oxygen evolution (OER) is limited by its slow kinetics (Zhou et al., 2018; Sultan et al., 2019). At present, precious metal is still the best catalyst (Li et al., 2018); in order to reduce the consumption of precious metal, looking for cheap alternatives is the general trend.

Recently, the transition metallic compound has attracted a lot of attention due to their intrinsically enhanced safety and high availability through the conversion reaction (Kim et al., 2021; Zhang et al., 2021; Zhu et al., 2021). Among them, single-component metallic compound has also shown excellent behavior as an electrocatalyst for water splitting (Jebaslinhepzybai et al., 2021). In particular, the introduction of the second metal ions can change the electronic state of the active metal, vacancy concentration (Yuan et al., 2021), coordination environment, or electron band structure (Zhang et al., 2017b), therefore enhancing HER kinetics (Yang and Chen, 2020; Li et al., 2021), such as the use of plasma treatment method to activate the Cu surface (Lee et al., 2018; Tomboc et al., 2020), by doping additional elements or with other metal alloys to adjust the binding energy of the reaction intermediates (Gatalo et al., 2019), and the Cu species into a specific structure or a specific crystal plane (Koh and Strasser, 2007; Liu et al., 2021; Yan et al., 2021; Yang et al., 2021). Christoph R. Muller (Kuznetsov et al., 2020) studied the surface oxygen vacancies (V_O_) in Y_1.8_M_0.2_Ru_2_O_7−δ_ (M = Cu, Co, Ni, Fe, Y) by an increased concentration of V_O_ sites correlating with a superior OER activity. These studies show that not only the dispersion of metal particles but also the properties of the substrate can alter the electronic states and chemical properties of the active site (Zhu et al., 2017), thus altering the catalytic activity (Yan et al., 2020; Zhang et al., 2020; Qiu et al., 2021; Wu et al., 2021). However, obtaining a catalyst of high activity remains a huge challenge. Therefore, it is necessary to design a new strategy for Cu-based catalysts.

Herein, M-Cu (M = Mn, Fe, Co, Ni, and Pt) multicomponent metallic compound materials are studied as electrocatalytic materials for water splitting. Different metal materials attached to the copper foam substrate can change the valence states of copper and oxygen, resulting in the change of electronic structure of the materials, thus changing its catalytic activity. As a result, for non-precious metal, the overpotentials for the Co–Cu sample at a current density of 10 mA/cm^2^ were 207.0 mV for OER and 329.8 mV for HER in 1 M KOH. Moreover, when adding the precious metal Pt, the high OER and HER catalytic efficiencies were also observed in the Pt–Cu sample.

## Experimental Section

The Cu foam was purchased from Jia Yisheng Co., Ltd. (Kun Shan); the thickness is 1.5mm, the surface density is 600 g/m^2^, and the hole number is 110 ppi. Firstly, the copper foam was cut into 2 cm × 2 cm sized squares and soaked in hydrochloric acid (deionized water: hydrochloric acid = 3:1) using an ultrasonic cleaner for 30 s.

Then, they were immersed in an equimolar solution of FeCl_3_ (0.6 g per 30 ml), CoCl_2_, NiCl_4_, MnCl_2_, and chloroplatinic acid solution for 30 min, separately. Finally, they were taken out and allowed to dry using a hair dryer.

## Material Characterization

The morphologies of the products were investigated by field-emission scanning electron microscopy (SEM, Helios Nanolab 600i), transmission electron microscope (TEM, Tecnai G2 F30), X-ray diffraction (XRD, D8 Advance), and X-ray photoelectron spectroscopy (XPS, Thermo Fisher).

## Electrochemical Test

All electrochemical performances were measured in the electrochemical workstation (CHI 760E). The HER and OER properties were measured in a three-electrode system, and the obtained samples, Hg/HgO, and carbon rod were used as working electrode, reference electrode and counter electrode, respectively. The electrolyte was 1.0 M KOH solution. All the potential was converted to RHE. LSV tests were carried out at a scan rate of 2 mV/s. The EIS measurement was carried out in a frequency range from 0.1 Hz to 100 kHz.

## Results and Discussion

**FIGURE 1:**
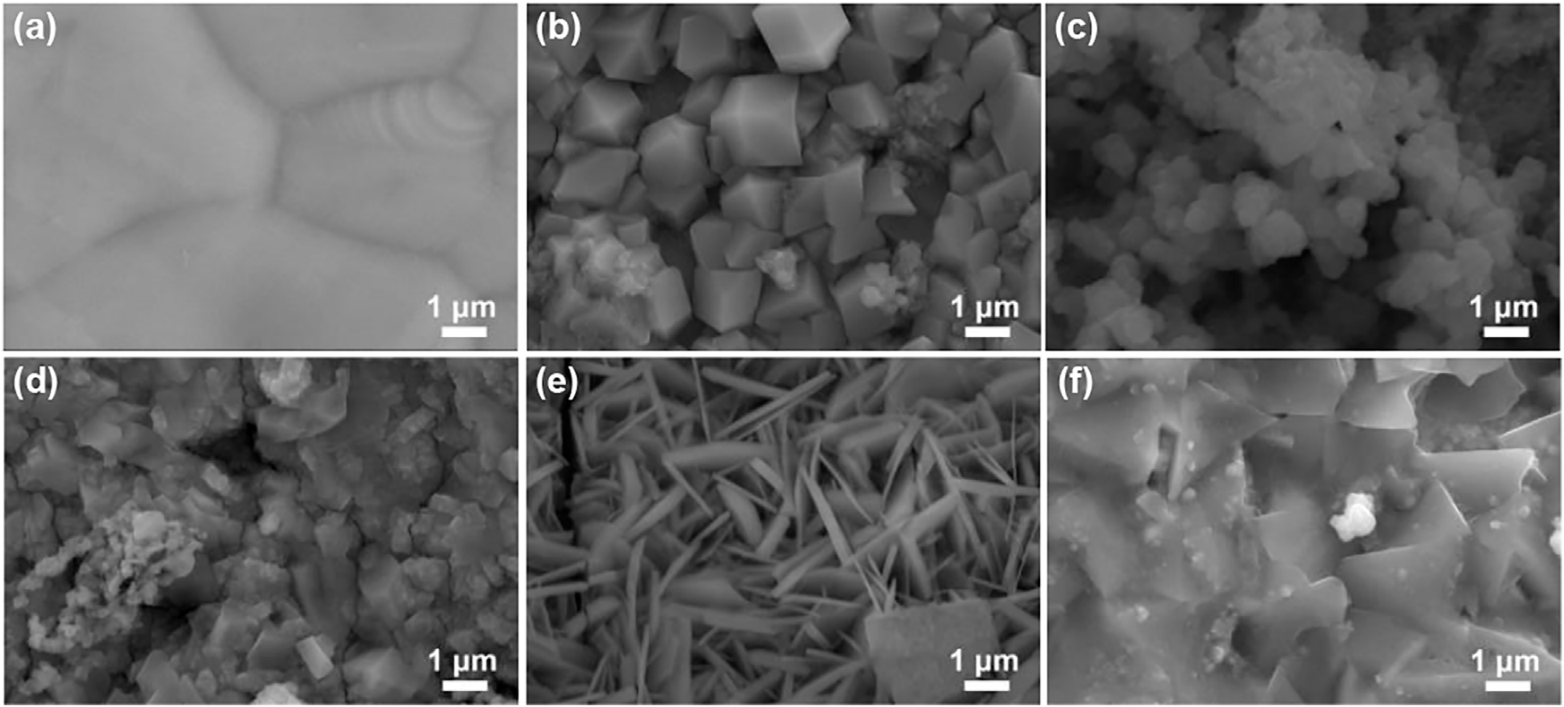
SEM images of **(A)** Cu foam, **(B)** Mn–Cu, **(C)** Fe–Cu, **(D)** Co–Cu, **(E)** Ni–Cu, and **(F)** Pt–Cu.

In this work, the M-Cu (M = Mn, Fe, Co, Ni, and Pt) samples were achieved by a facile method; the surface morphology of the pristine samples was investigated by SEM. As shown in Figure 1, combining the SEM-mapping analysis (Supplementary Figure S1), the simple electron microscopic diagram proves that the whole foam copper is covered by the sample.

X-ray diffraction (XRD) analysis is employed to confirm the crystal structure of the as-synthesized M-Cu samples. It can be seen from Supplementary Figure S2 that the M-Cu samples were located at 43.3, 50.4, and 74.1°,corresponding to (111), (200), and (220) crystal planes of the spinel Cu (PDF# 04-0836), and the peaks located at 37.0, 42.6, 62.4, and 74.4°, corresponding to (111), (200), (220), and (311) crystal planes of the spinel Cu2O (PDF# 34-1354). Obviously, no other diffraction peaks appeared.

**FIGURE 2:**
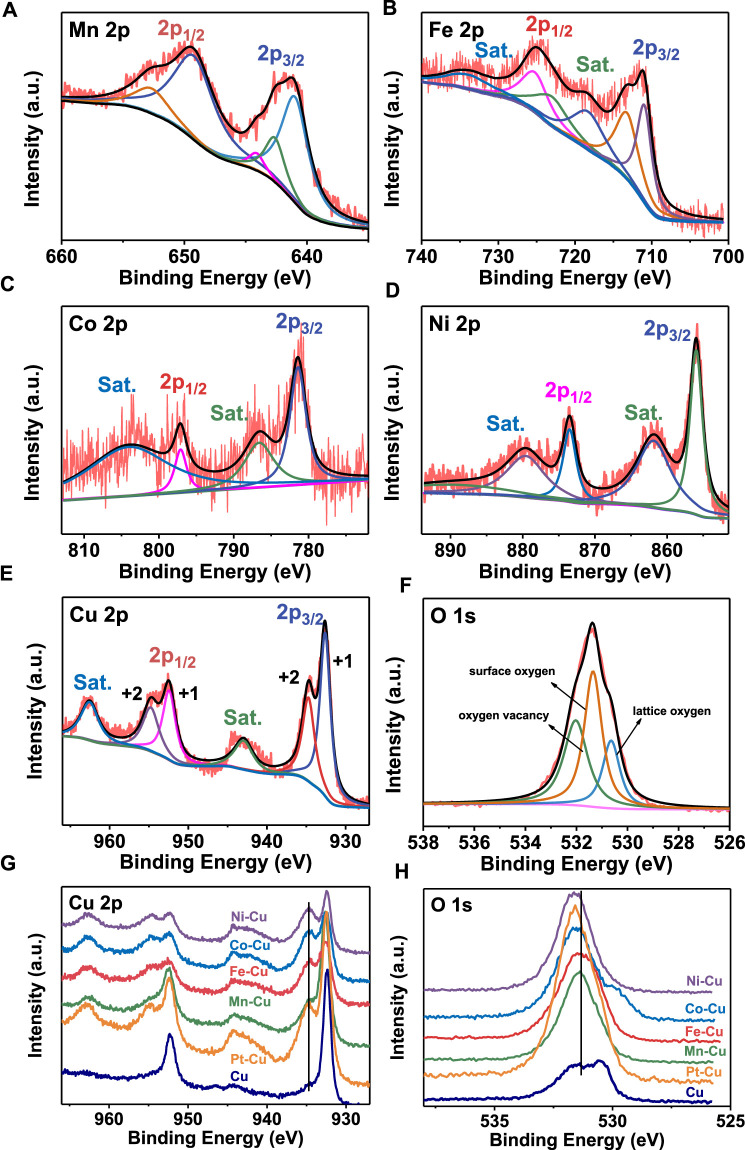
XPS spectra of **(A)** Mn 2p for Mn–Cu, **(B)** Fe 2p for Fe–Cu, **(C)** Co 2p for Co–Cu, **(D)** Ni 2p for Ni–Cu and XPS spectra of **(E)** Cu 2p and **(F)** O 1s for Pt–Cu, **(G)** Cu 2p and **(H)** O 1s for all M-Cu samples.

The surface composition and chemical states of the samples were further explored by XPS, as shown in Figure 2. It shows that the main elements of Mn, Fe, Co, and Ni were recorded from the XPS. Figure 2A depicts the peaks at 640.9, 642.6, 649.2, 644.1, and 652.7 eV, assigned to two spin–orbit doublets of Mn 2p_1/2_ and Mn 2p_3/2_ and two shake-up satellite peaks (Guo et al., 2017). Figure 2B shows the Fe 2p spectrum consisting of two spin–orbit doublets and two shake-up satellites. The multiple peaks at 713.2, 725.2, 711.0, and 713.1 eV can be assigned to the Fe^3+^ 2p_1/2_, Fe^3+^ 2p_3/2_, Fe^2+^ 2p_1/2_, and Fe^2+^ 2p_3/2_, while the shake-up satellite peaks are observed at 718.3 and 733.9 eV (Ge et al., 2018). The high-resolution Co 2p spectrum (Figure 2C) displays two major peaks at 781.4 and 797.1 eV, corresponding to Co 2p_3/2_ and Co 2p_1/2_, respectively (Wang et al., 2021; Tabassum et al., 2019). The Ni 2p XPS spectra can be fitted to four components located at 855.9 and 873.5 eV corresponding to Ni 2p_3/2_ and Ni 2p_1/2_, as shown in Figure 2D (Wu et al., 2017).

The valence state of the Cu and O elements for all M-Cu (M = Mn, Fe, Co, Ni, and Pt) was studied to establish its potential correlations with the HER and OER activities. It can be seen that compared with the pure copper foam, the valence states of copper and oxygen elements changed obviously after adding other metal elements from Figure 2. Three fitting components were found on the surface of the sample by O 1s XPS detection: lattice oxygen, surface oxygen species, and oxygen vacancies at the respective energies of ∼530.6, 532.3, and 531.3 eV (Kuznetsov et al., 2020)^,^ (Banger et al., 2011). However, for the Cu 2p spectrum, which consists of two spin–orbit doublets and two shake-up satellites, the multiple peaks at 932.9, 934.7, 952.4, and 954.8 eV can be assigned to the Cu^+^ 2p_3/2_, Cu^2+^ 2p_3/2_, Cu^+^ 2p_1/2_, and Cu^2+^ 2p_1/2_, respectively (Chauhan et al., 2017; Zhang et al., 2019b), while the shake-up satellite peaks are observed at 934.0 and 962.5 eV. After the addition of the second metal ion, the second metal ion will be doped into Cu_2_O, which may change the lattice parameters, resulting in the shift of the peaks. This indicates that the strong electronic interaction between the cations and the second metal ion leads to electron accumulation around Cu centers (Yan et al., 2021). And for O 1s, the addition of the second metal ion results in the positive shift of the peaks. This indicates that different oxygen vacancy concentrations existed in M-Cu samples (Kuznetsov et al., 2020). Obviously, with the addition of the second metal ion, the valence states of copper and oxygen are changed in different degrees, which will have different degrees of influence on the HER and OER performances.

**FIGURE 3:**
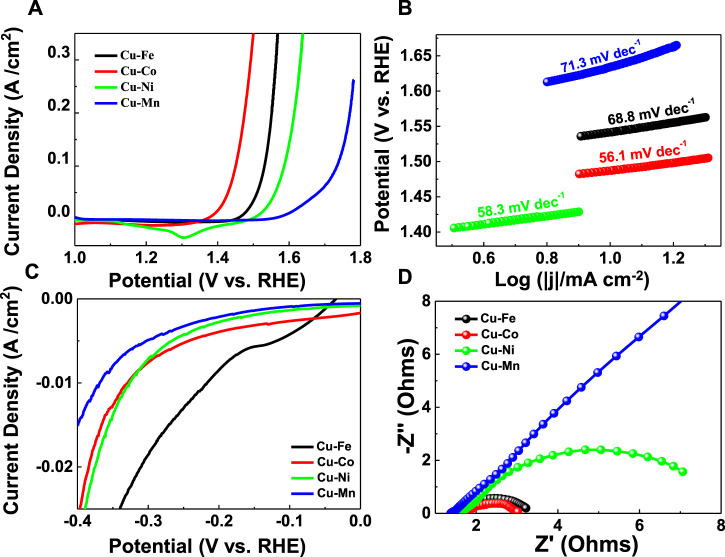
**(A)** LSV curves and **(B)** Tafel plots of the obtained catalysts for HER tests. **(C)** LSV curves of the obtained catalysts for OER tests. **(D)** Nyquist plots of the obtained catalysts.

The HER and OER performances of M-Cu (M = Mn, Fe, Co, Ni, and Pt) samples were measured in 1 M KOH solution using a conventional three-electrode electrochemical setup. By contrast, the HER and OER performances of Cu foam have been tested and are shown in Supplementary Figure S3. The overpotentials for the Cu foam at a current density of 10 mA/cm^2^ were 728.0 mV for OER and 438.1 mV for HER in 1 M KOH. The higher OER catalytic efficiency is observed on the Co–Cu with a low overpotential of 207.0 mV at 10 mA/cm^2^, as evident in Figure 3A, smaller than Fe–Cu: 256.8 mV, Ni–Cu: 309.8 mV, and Mn–Cu: 403.2 mV. The excellent kinetic performance of Co–Cu can be proved by its smallest Tafel slope of 56.1 mV/dec (Figure 3B). As depicted in Figure 3C, it can be found that the HER activity is also with a low overpotential of 215.8 mV at 10 mA/cm^2^ for the Fe–Cu sample and 329.8 mV at 10 mA/cm^2^ for the Co–Cu sample. The excellent electrochemical performance of all samples can be attributed to the improvement of fast charge transfer kinetics (Yan et al., 2022), so electrochemical impedance spectroscopy (EIS) as a further research was carried out. As shown in Figure 3D, the charge transfer resistance of Co–Cu is also significantly smaller, illustrating the small electrode polarization and better electrochemical kinetics. In order to explore the stability of the obtained samples, the chronoamperometric HER test is conducted in Supplementary Figure S4. The steady curves obtained at -10 mA cm^−2^ suggest the stable hydrogen evolution behaviors. Additionally, we have added the electrochemical active area (ECSA) tests for the samples (Supplementary Figure S5), through the following equation (Voiry et al., 2018):

ECSA = CdlCs

where C_dl_ represents the electrical double-layer capacitance of the corresponding catalyst and C_s_ represents the specific capacitance of smooth oxide in 1 M KOH, which is about 0.04 mF/cm^2^.

The calculated double-layer capacitance results of Co–Cu, Fe–Cu, Ni–Cu, and Mn–Cu were 20.3, 11.7, 13.4, and 18.7 mF cm^−2^, respectively. We have also standardized the polarization curves into TOF as follows (Zhang et al., 2017c):

TOF_O2_ = |J| ^∗^ mA/ECSA ^∗ ^1 C s^−1^/1000 mA ^∗ ^1 mol e^–^/96,495.3 C ^∗ ^1 mol O_2_/4 mol e^–^^∗ ^6.022 ^∗ ^10^23^ O_2_ molecules/1 mol O_2_

= |J|/ECSA ^∗ ^1.56^∗^10^15^ O_2_ s^−1^ per mA/cm^2^

The calculated results are shown in Supplementary Figure S6 as follows: in the overpotential of 300 mV, TOF values of Co–Cu, Fe–Cu, Ni–Cu, and Mn-Cu were 1.928^∗^10^15^, 0.516^∗^10^15^, 0.079^∗^10^15^, and 0.002^∗^10^15^ O_2_ s^−1^ per mA/cm^2^, respectively.

**FIGURE 4:**
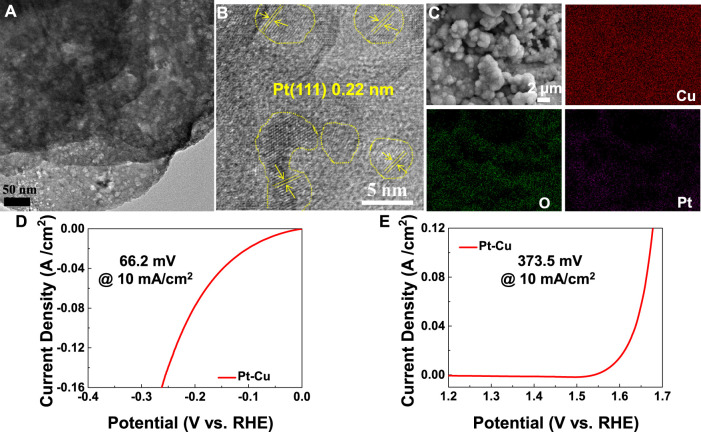
**(A)** TEM image and **(B)** HRTEM image of Pt–Cu. **(C)** Element mapping of Pt–Cu. LSV curves of **(D)** HER tests and **(E)** OER tests for the Pt–Cu sample.

Further insights into the morphology and structure of the as-prepared Pt–Cu products were elucidated by TEM and HRTEM. The (111) planes of Pt were observed in the HRTEM images of Figure 4B. Distribution of elements across Pt–Cu was analyzed using the high-resolution EDX elemental mapping analysis in transmission electron microscope (TEM). These EDX maps (Figure 4C) confirm the uniform distribution of Pt, Cu, and O across the sample. The Pt 4f peak (Supplementary Figure S7) could be separated into two peaks: 75.1 and 77.5 eV, representing the Pt 4f_5/2_ and Pt 4f_7/2_, respectively (Han et al., 2020). Moreover, when it comes to precious metal Pt, the high OER and HER catalytic efficiencies are observed on the Pt–Cu, as evident in Figures 4D,E.

## Conclusion

In summary, we have synthesized the M-Cu (M = Mn, Fe, Co, Ni, and Pt) samples by a facile method on a copper foam substrate. The electrode shows good HER and OER performances. For non-precious metal, the overpotentials for the Co–Cu sample at a current density of 10 mA/cm^2^ were 207.0 mV for OER and 329.8 mV for HER in 1 M KOH solution. Moreover, when adding precious metal Pt, the high OER and HER catalytic efficiencies were also observed in the Pt–Cu sample. The good HER and OER performances come from the introduction of the second ions, which change the valence states of copper and oxygen elements, resulting in the change of electronic structure of the materials, thus changing its catalytic activity.

## Data Availability

The original contributions presented in the study are included in the article/Supplementary Material; further inquiries can be directed to the corresponding author.
