# Supplementary material for: Cu-Based Multicomponent Metallic Compound Materials as Electrocatalyst for Water Splitting
Source: Front Chem. 2022 Jun 13;10:913874. doi: 10.3389/fchem.2022.913874 (PMC9234134; doi:10.3389/fchem.2022.913874)
Supplement: Supplementary file 1 [file Table1.DOCX]

Support information for:

**Cu-based multicomponent metallic compound materials as electrocatalyst for** **water splitting**

*Peijia Wang^#^, Jingjing An ^#^, Zhenyu Ye and Xiaohang Zheng**

*School of Materials Science and Engineering, Harbin Institute of Technology, Harbin, 150001, China.*

*Corresponding authors. E-mail addresses: zhengxiaohang@hit.edu.cn.

#These authors contributed equally to this work.

Fig.S1 EDS-Mapping diagram of M-Cu samples: (a) Mn-Cu, (b) Fe-Cu, (c) Co-Cu and (d) Ni-Cu.

Fig.S2 XRD patterns of M-Cu samples.

Fig.S3 LSV curves of (d) HER tests and (e) OER tests for the Cu foam.

Fig.S4 Chronopotentiometry measurements for (a) Co-Cu and (b) Pt-Cu in HER.

Fig. S5 CV curves of (a) Co-Cu, (b) Fe-Cu, (c) Mn-Cu and (d) Ni-Cu in the non-faraday potential region; (e) the calculated electrical double-layer capacitor (C_dl_) values.

Fig. S6 TOF curves of M-Cu samples

Fig. S7 XPS spectra of (a) Cu 2p, (b) Pt 4f and (c) O 1s for Pt-Cu sample.
